# Microbial contamination of water intended for milk container washing in smallholder dairy farming and milk retailing houses in southern Ethiopia

**DOI:** 10.1186/s40064-016-2841-x

**Published:** 2016-07-28

**Authors:** Kebede Amenu, Desalew Shitu, Mesele Abera

**Affiliations:** School of Veterinary Medicine, Hawassa University, P. O. Box 5, Hawassa, Ethiopia

**Keywords:** Smallholder dairy, Water quality, Milk hygiene, Post-supply contamination, Wash water, Milk retail

## Abstract

The water used during handling and processing of milk products can be potential sources of microbial contamination with possible negative consequences on food safety. Especially, the water used in keeping the hygiene of milking and milk storage utensils is crucial to keep the quality and safety of the products. Therefore, the current study was designed to assess the bacteriological quality of water used for cleaning milking and milk storage equipment in smallholder dairy production in Hawassa and its surroundings. A total of 79 water samples were collected: 26 from milk collecting houses in Hawassa and 53 from selected smallholder dairy farms (Hawassa = 14, Arsi Negele = 29 and Yirgalem = 10). Out of the total samples, 18 samples were collected directly from pipe and 61 from storage containers (46 from narrow opening and 15 from wide opening containers). The overall prevalence of *E. coli* exceeding zero CFU/ml was 39.2 %. From analyzed samples, high prevalence of positive samples for *E. coli* was found in water samples taken from wide opening containers (66.7 %). A number of bacteria were isolated and presumptively identified which include *Bacillus* sp. 6.3 % (n = 5), *Citrobacter* sp. 1.3 %(n = 1), *E. coli* 39.2 % (n = 31), *Enterobacter* sp. 2.5 % (n = 2), *Klebisella* sp. 7.6 % (n = 6), *Micrococcus* sp. 6.3 % (n = 5), *Pseudomonas* sp. 6.3 % (n = 5), *Staphylococcus aureus* 6.3 % (n = 5), *Staphylococcus epidermidis* 13.9 % (n = 11) and *Streptococcus* sp. 1.3 % (n = 1). The bacteriological quality of water especially, water stored in household storage containers in present study area was found to be contaminated with different bacteria indicating potential food safety problem and health risk to the society. In this respect, people handling water should be educated on its proper handling and the risk of contamination during storage. To minimize contamination, materials with narrow mouth and lid should be used. Further study is recommended on the relationship between the bacteriological quality of water and the behavior of water users.

## Background

Inadequate access to safe drinking water is one of the major health problems in many developing countries and responsible for the high morbidity and mortality of people (Ashbolt 2004). Poor quality water is responsible for high prevalence of diarrheal diseases especially in children. People can be exposed to waterborne pathogenic agents either through drinking contaminated water or when the contaminated water is used for food production and/or processing (Kirby et al. 2003). Since milk and dairy products are rich sources of nutrients such as proteins, fats and minerals they are highly prone to microbial contamination and spoilage (Fernandes 2009). Consequently, the water used during handling and processing of milk products can be potential sources of microbial contamination for milk and dairy products with consequences on food safety (Amenu et al. 2014).

Cleaning of equipment used in the milk storage and transportation is highly crucial activity to minimize microbial contamination of milk and subsequently to improve its microbiological safety (O’Connor 1995). In this regard, it is recommended that water used in cleaning of equipment and processing of milk should have quality standard equivalent to drinking water (Terplan 1980). However, in most developing countries like Ethiopia, smallholder dairy farmers and informal milk retailers may not have access to water source of such a good quality or if so, contamination can be a problem during handling or storage of water (Amenu 2013). Consequently, poor quality water can be a major contributing factor for the low quality and safety of milk and milk products. Microbial contamination of drinking water during handling or storage after collection is often significant problems in many developing countries where water transportation and storage for later use is a common practice (Wright et al. 2004). Similarly, the microbiological quality of water destined for washing and cleansing of milk equipment can be seriously affected by the handling and storage conditions.

The microbiological quality of food or water is often assessed using indicator organisms whose concentration or density is usually associated with health risks (WHO 2011). Detection and enumeration of *Escherichiacoli* represents a very specific and well-accepted microbiological quality indicator of food or water (Edberg et al. 2000). Some studies revealed that wash water can be source of bacterial contamination for milk and further compromise the quality and safety of milk or milk products (Kivaria et al. 2006; Perkins et al. 2009).

In this regard, quality of water used by smallholder dairy farmers should be assessed to protect the public from the related adverse health effects. Therefore, the objective of the present study was to evaluate the microbiological quality of water used for washing containers in smallholder dairy production based on *E. coli* count and qualitative presumptive isolation of bacterial contaminants.

## Methods

### Study area

**Fig. 1:**
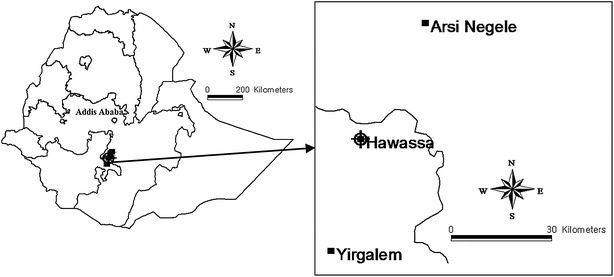
Map of the study area

The study area encompassed urban environs of Arsi Negele, Hawassa and Yirgalem (Fig. 1) located at about 231, 270 and 315 km south of Addis Ababa, respectively. Water samples were collected from smallholder dairy farmers and milk retail shops. Hawassa is capital city of South Nations and Nationalities People Regional State (SNNPRS) with an estimated human population of 316,842. In the study area, raw milk is collected from smallholder farmers of the milkshed by milk retailers and again sold to consumers without further processing. In some cases, the raw milk is processed to produce traditional dairy products such as *ergo* (curd milk), *ayib* (soft cheese) and butter. Recently, few milk pasteurization plants are also emerging in the area.

### Study design

A cross-sectional microbiological quality assessment of water used in hygienic measures related to milk handling and processing in milk retail shops and dairy cattle keeping households was carried out. Milk collecting and retail shops in Hawassa were asked for their cooperation and accordingly a total of 26 of them were identified. Thereafter, 53 smallholder dairy farmers supplying milk to the collectors/retailers were identified and traced back to collect water samples. Overall, a total of 79 water samples to be used for cleansing and washing of milking and milk storage containers were collected (26 from milk retail shops, 53 from the houses of smallholder dairy farmers).

### Water sample collection and transportation

The water samples were collected from milk collecting houses and selected individual households with dairy farm. The initial source of water was pipe-borne for all milk collecting and individual smallholder farming households. A water sample of 100 ml was collected from smallholder dairy farms. The samples were collected from directly from pipe (18), from narrow mouthed containers (46), and from wide mouthed containers (15) (e.g. barrels, plastic buckets and jugs). In order to simulate the actual water use practices in the area, no sterilization of the pipe faucet was carried out. The samples were collected in sterile capped plastic containers by following strict aseptic procedures. The water samples were kept in an ice-box and transported to Hawassa University, Veterinary Microbiology Laboratory within 6 h of collection for bacteriological analysis.

### Bacteriological analysis

#### Enumeration of *E. coli*

**Fig. 2:**
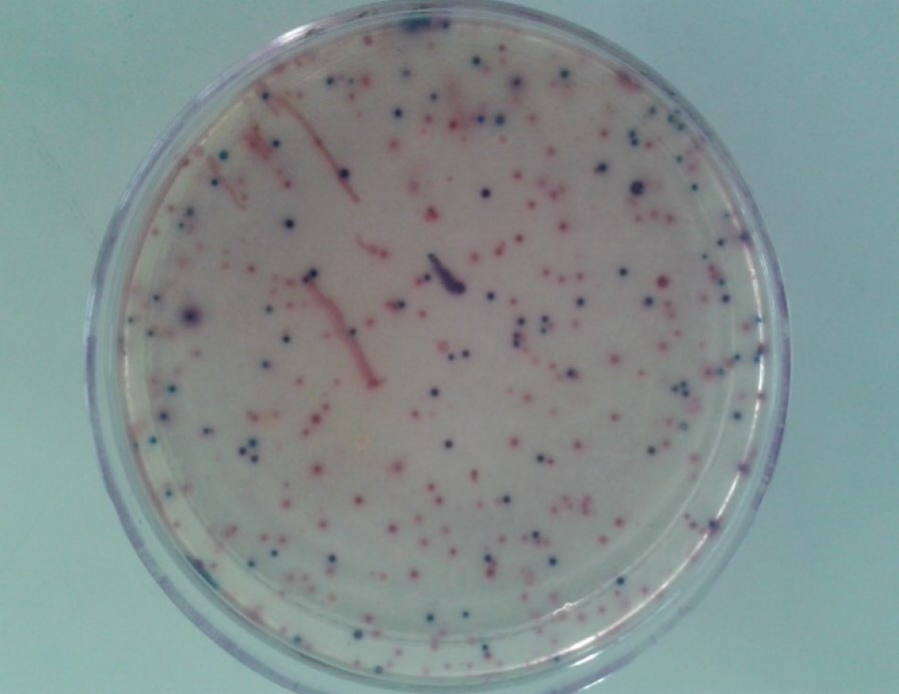
Purple and pink colonies on Brilliance *E. coli*

Enumeration of total coliforms and *E. coli* in water samples were carried out using commercially available chromogenic medium, Brilliance™ *E. coli*/coliform selective agar (Oxoid, CM1046). The agar was prepared according to manufacturer instruction and the molten medium was kept at +50 °C in a water bath. After that, 1 ml of the water sample was pipetted into an empty sterile Petri dish after being thoroughly mixed and covered with approximately 15–20 ml of the molten medium. The plates were gently swirled thoroughly to mix the water sample and the medium and incubated for 24 h at 37 °C. After 24 h incubation, purple and pink colonies were counted as *E. coli* (Fig. 2) and expressed as colony forming units (CFU)/1 ml of water.

#### Bacterial isolation and presumptive identification

**Fig. 3:**
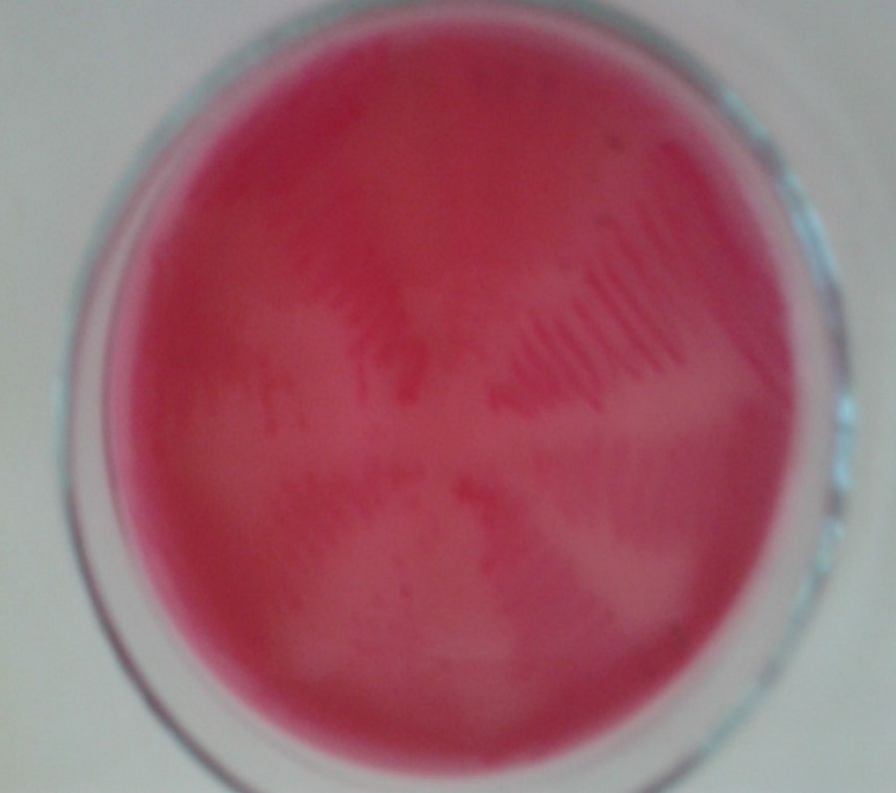
Lactose fermentation on MacConkey agar

**Fig. 4:**
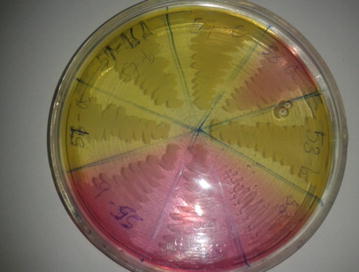
Yellow and pink fermentation on mannitol salt agar (*S. aureus* and *S. epidermidis,* respectively)

**Fig. 5:**
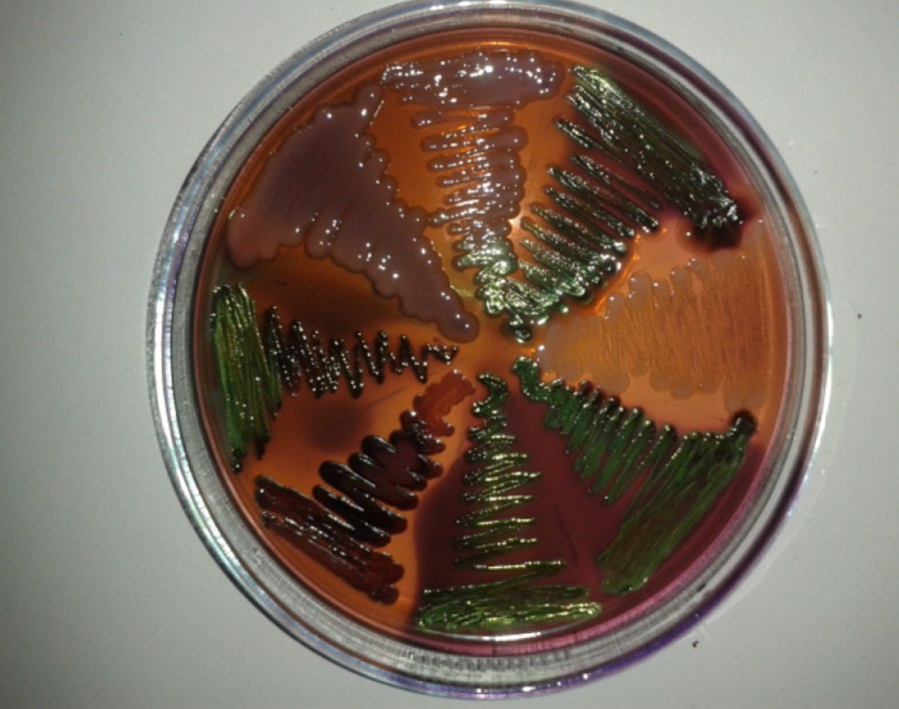
Growth *of E. coli* on EMB agar with metallic sheen

Isolation of bacteria was conducted by inoculating water sample simultaneously into sheep blood agar and MacConkey agar. After incubation for 24 h, individual bacterial colonies were observed and sub-cultured into new blood agars to get pure colonies. Growth of bacteria on MacConkey agar was characterized by its color on the media (e.g., whether fermenting lactose or not) (Fig. 3). The colononies from blood agar were stained with Gram stain and assessed whether positive or negative, for cellular morphology (rod or coccus) and cellular array (pair, cluster or chain) using a compound microscope. After this, different biochemical tests were done for presumptive identification of the bacterial isolates to genus or species level following standard methods (Quinn et al. 1999). Biochemical tests employed for the presumptive identification of the isolates were catalase test, culture on mannitol salt agar (Fig. 4), motility, indole, methyl red, Voges-Proskauer, citrate (IMViC) and triple sugar iron (TSI) reaction tests. Isolates *E. coli* were confirmed by growing on Eosin Methylene Blue (EMB) agar in which the isolates showed metallic green sheen appearance (Fig. 5).

### Data analysis

The collected data was entered into Microsoft Excel and the difference in the proportion of *E. coli* positive samples among different sampling units was compared using Chi-sqaure test. Descriptive statistics (e.g. frequency, proportion) also calculated.

## Results

### *E. coli* count

Microbiological contamination of water sampled from different points is shown in Table 1. The load and occurrence *E. coli* was generally higher in water samples taken from wide mouthed containers compared with narrow mouthed containers. The difference in the proportion of *E. coli* contaminated between different sampling points was statistically significant.

**Table 1:** Contamination of water samples taken from pipe, narrow and wide opening containers with *E. coli*

Sampling points	N positive	% positive*	Mean	Median	IQR**
Directly from pipe (n = 18)	1	5.6	3.0	3.0	0
Narrow mouth container (n = 46)	20	43.5	8.0	6	12.5
Wide mouth container (n = 15)	10	66.7	8.5	6.5	8.0
Total	31	39.2	8.0	6.0	12.0

* Chi square = 13.64, *p* value = .001; ** *IQR* interquartile range

### Bacteria isolates

Out of the overall water samples analyzed in the present study (n = 79), in about 80 % of the samples one or more bacteria isolates were detected. In majority of the cases, the highest detection of bacteria was found in water samples obtained from wide opening containers. In this regard, water samples collected directly from pipe was superior in its bacteriological quality compared to the water samples stored containers indicating post-supply contamination. One or more bacteria were isolated from all of the water samples stored in wide opening containers. The bacteria isolated and identified from the water samples were *Bacillus* sp., *Citrobacter* sp., *Enterobacter* sp., *Klebsiella* sp., *Micrococcus* sp., *Pseudomonas* sp., *Staphylococcus aureus, Staphylococcus epidermidis, Streptococcus* sp. and unidentified bacteria (Table 2).

**Table 2:** Percentage of bacterial isolates (other than *E. coli*) from water samples collected from different points (directly from pipe, narrow mouth and wide mouth containers)

Microorganism	Total (n = 79)	Directly from pipe (n = 18)	Narrow (n = 46)	Wide (n = 15)
*E. coli*	39.2	5.6	43.5	66.7
*Bacillus* sp.	6.3	0	10.9	0
*Citrobacter* sp.	1.3	0	0	6.7
*Enterobacter* sp.	2.5	0	2.2	6.7
*Klebsiella* sp.	7.6	5.6	4.5	20.0
*Micrococcus* sp.	6.3	0	10.9	0
*Pseudomonas* sp.	6.3	11.1	4.4	6.7
*Staphylococcus aureus*	6.3	5.6	6.5	6.7
*Staphylococcus epidermidis*	13.9	5.6	10.9	33.3
*Streptococcus* sp.	1.3	0	2.2	0

Narrow = narrow mouth container; wide = wide mouth container

## Discussion

Microbial contamination of water during handling and storage after collection is one of the major problems in the management of water destined for domestic use in sub-Saharan Africa (Harris et al. 2013). Water for domestic use is stored in houses before drinking. This is due to the fact that the sources water for domestic use is located far apart from the houses of the people or water supply is interrupted. Multiple factors are responsible for the microbiological quality deterioration of water intended for drinking and other domestic uses (Trevett et al. 2004). The present study showed that most of the samples collected and analyzed from wide opening containers were high for *E. coli* count and other bacterial contaminants compared to the samples from narrow opening containers. Such high levels of microbial contamination and impaired microbiological quality in wide opening storage containers may be associated with the vulnerability of introducing hands and cups during water use. Similar studies have also noted the vulnerability of storage containers with such wide opening to fecal and other contamination (Oswald et al. 2007). According to Clasen et al. (2003), use of narrow mouth storage containers with valve can minimize microbial contamination and subsequently reduce the incidence of waterborne diseases. Simultaneously, considerable number of water samples collected from narrow opening container was found to be contaminated with *E. coli* in the present study. This could be due to the narrow opening of the container can prevent its effective cleaning after every use and microbial build up can occur. Another justification for this increase may be due to the fact at the time of the sample collection, most of the storage containers were open and even in some households they were outside in open environment which can increase the chance of contamination of stored water resulting microbial build up.

In present study, variety of bacterial genera including *E. coli*, *Klebsiella* sp., *Citrobacter* sp., *Enterobacter* sp., *Pseudomonas* sp., *Micrococcus* sp., *Staphylococcus* sp., and *Streptococcus* sp. and *Bacillus* sp. were detected in the water samples. Some of the isolated bacteria in the present study can be opportunistically pathogenic to humans and responsible for health problems such as urinary tract infection, diarrhea and ear infections. Use of contaminated water in the handling and processing of milk products can cause a higher potential health risk than the risk through direct drinking. This is due to the fact that multiplication of pathogenic micro-organisms can occur in milk and milk products with amplification of the load of the pathogens (Amenu 2013).

In conclusion the present study showed high microbial contamination of water after supply due to unhygienic practices during collection and storage. In this respect, persons handling water should be educated on the proper handling and the risk of post-collection contamination. If possible, materials with optimally narrow mouth allowing effective washing and lid should be used. Moreover, further study should be carried out on the relationship between bacteriological quality of water and the practices and behavior of the water users.
